# IMPROVING BRAIN AGE PREDICTION MODELS: INCORPORATION OF AMYLOID STATUS IN ALZHEIMER’S DISEASE

**DOI:** 10.1093/geroni/igz038.347

**Published:** 2019-11-08

**Authors:** Maria Ly, Nishita Muppidi, Helmet Karim, Gary Yu, Akiko Mizuno, William Klunk, Howard Aizenstein

**Affiliations:** University of Pittsburgh, Pittsburgh, Pennsylvania, United States

## Abstract

Brain age prediction may serve as a promising, individualized biomarker of brain health and may help us understand the heterogeneous biological changes that occur in aging. Brain age prediction is a machine learning method that estimates an individual’s chronological age from their neuroimaging scans. If predicted brain age is greater than chronological age, that individual may have an “older” brain than expected, suggesting that they may have experienced a higher cumulative exposure to brain insults or were more impacted by those pathological insults. However, contemporary brain age models include older participants with amyloid pathology in their training sets and thus may be confounded when studying Alzheimer’s disease (AD). We showed that amyloid status is a critical feature for brain age prediction models by training a model on 808 individuals without significant amyloid pathology from the ADNI, OASIS-3, and IXI cohorts. Our model accurately predicted brain age in the training and independent test sets, comparable to previous published models: [r(807) = 0.94, R2 = 0.88, p=0.001, MAE = 4.9 years, p=0.001], [r(39) = 0.67, R2 = 0.45, and MAE = 4.6 years]. We demonstrated significant differences between AD diagnostic groups [F(3,431)=30.7, p<0.001], and our model was the first to delineate significant differences in brain age relative to chronological age between cognitively normal individuals with and without amyloid [mean difference, 95% CI; CN-Aβ(-) (-3.4, -4.9:-1.8), CN-Aβ(+) (-0.7, -1.9:0.5)]. Ultimately, incorporation of amyloid status in brain age prediction models improves the utility of brain age as a biomarker for aging and AD.

